# Hartmann's Reversal: Controversies of a Challenging Operation

**DOI:** 10.1155/2022/7578923

**Published:** 2022-11-09

**Authors:** Claudia Reali, Kalle Landerholm, Bruce George, Oliver Jones

**Affiliations:** ^1^Department of Colorectal Surgery, Coventry University Hospital, Clifford Bridge Road, Coventry CV2 2DX, UK; ^2^Department of Surgery, Ryhov Country Hospital, Jonkoping, Sweden; ^3^Department of Colorectal Surgery, Oxford University Hospitals, Old Road, Headington, Oxford OX3 7LE, UK

## Abstract

**Purpose:**

Hartmann's reversal is a complex operation with a high morbidity rate. Minimally invasive surgery has been used to reduce the impact of surgery on fragile patients. The aim of this comparative study is to look at the results of Hartmann's reversal procedures with different approaches.

**Methods:**

All the patients who underwent Hartmann's reversal were collected retrospectively (124 cases). Sixty-four patients (50.4%) had an open operation, 6 cases (5%) were treated with a conventional laparoscopic approach, 34 patients (28.1%) underwent single incision laparoscopic surgery (SILS), and 20 (16.5%) required other additional trocars.

**Results:**

SILS operations were slightly longer than the open procedures (175 min vs 150 min), with the same rate of postoperative complications and reoperations (*p* = 0.83 and *p* = 0.42), but with a shorter hospital stay (5 days *p* = 0.007). Age (*p* = 0.03), long operative time (*p* = 0.01), and ASA score (*p* = 0.05) were identified as independent factors affecting postoperative morbidity. The grade of adhesions caused a longer operative time (*p* = 0.001) and a higher risk of conversion (*p* < 0.001), and short rectal stump increased the risk of protective loop ileostomy (*p* = 0.008). Patients with grade 2-3 of adhesions had a longer length of stay (*p* = 0.05).

**Conclusions:**

Minimally invasive procedures had a shorter hospital stay and did not show any increase in morbidity rate when compared with open cases. Age, longer operative time, and ASA score increased the risk of postoperative complications. Furthermore, patients with a short rectal stump had a higher chance of having a defunctioning ileostomy.

## 1. Introduction

Hartmann's reversal (HR) is a challenging operation because of the high morbidity rate (30–60%) and unpredictable operative findings [1]. Even though Hartmann's procedure (HP) has been considered a safe choice in an emergency, the stoma formation is often permanent due to comorbidities which prevent future colostomy closure [2]. In fact, only 50–60% of patients have bowel continuity restored [2–6].

An open approach to reversing a colostomy is a big undertaking for many elderly patients, and the recovery is often affected by the postoperative pain as they cannot mobilise easily. On the other hand, a minimally invasive approach is technically difficult mostly due to adhesions and requires good laparoscopic skills. A single port approach, however, can be even more challenging due to the clashing of instruments very close to each other that causes less freedom of movement, the uncomfortable angulation, and the fewer ports that can be used [7, 8].

Many authors have tried to address this problem by comparing the laparoscopic approach to the traditional open one. Most of the results showed a reduction in morbidity with the minimally invasive surgery, considering it safe [2–5]. Despite that, not many articles have been published on single-port surgery through the stoma site.

The aim of this study is to identify the role of single incision laparoscopic surgery (SILS) through the stoma site in HR procedures and analyse the results of the different techniques. Furthermore, we analysed the factors that may contribute to postoperative morbidity and cause operative challenges. Predicting those factors can improve patient selection, improve outcomes, and reduce the chance of conversions.

## 2. Materials and Methods

All the patients of a single tertiary colorectal centre undergoing HR between 2010 and 2016 were identified through an online system, and the information concerning demographic, preoperative, operative, and postoperative details was organised in a database. Reviewing medical records was sometimes necessary in order to verify acquired information or gather further relevant data.

The following variables were analysed:

- Preoperative: this includes demographic details, indication for HP and urgency, previous abdominal surgical operations, and time elapsed from the primary operation to the reversal.
- Operative: this includes American Society of Anesthesiologists (ASA) Score, rectal stump length, duration of operation, amount of adhesions, operative complications, open or laparoscopic approach, number of patients converted to open surgery, causes of conversions, and combined operations.
- Postoperative: this includes morbidity, mortality, reoperation rate within 30 days postoperatively, hospital length of stay, and anastomotic leak.

All the procedures were carried out by 5 experienced colorectal laparoscopic surgeons who had completed their learning curve. An attempt to the single glove port approach was the standard, and the decision to perform an open operation was taken when other procedures were required at the same time (e.g., incisional hernia repair) or other complicating factors were identified (e.g., extremely dense adhesion during Hartmann's procedure). The stoma was mobilised, and the feasibility of a single glove port approach was considered. An Alexis wound protector was placed at the stoma site, and a glove was applied to it instead of the usual cover. The 3 ports with the respective laparoscopic instruments and camera were then placed through an opening at the fingers of the gloves. This allowed more freedom of movement and a reduction of costs. This approach has been already described in the literature with good results [9, 10]. All the anastomoses were formed end-to-end and stapled with a circular device. As per the communal agreement, all the patients that were deemed fit for reversal had the splenic flexure mobilised during the HP in order to facilitate the laparoscopic reversal. A loop ileostomy was formed to protect a low anastomosis or in the case of a positive air-leak test.

All patients who were 18 years of age or older and underwent Hartmann's reversal were included in the study.

Patients were divided into 3 groups: open, classic laparoscopic, and SILS. Some of the cases in the SILS group needed additional ports as the adhesiolysis was complex, and further trocars for a better retraction and accessibility were necessary to complete the operation. The difference between the laparoscopic and SILS groups was in the position of the camera. In the latter, the camera was placed in the glove port (making the operation more challenging because it was close to the other two instruments). Laparoscopic and SILS operations will be considered minimally invasive procedures.

The severity of adhesions was divided into four groups through an ad hoc classification:

- Grade 0 means no adhesions
- Grade 1 means few and soft adhesions
- Grade 2 means many adhesions that do not prevent a laparoscopic approach
- Grade 3 means hostile abdomen with dense adhesions that prevents a laparoscopic approach and tracer placement.

We further divide the adhesion in low grade (Grade 0 and 1) and high grade (grade 2 and 3) during the statistical analysis. As the severity of the adhesion was always documented in the operation notes, patients were included in one of the aforementioned groups.

The length of the rectal stump was defined in this article as long or short depending on whether it was above or below the peritoneal reflection. This was assessed intraoperatively during the section of the distal rectal stump at the time of Hartmann's procedure.

In the inflammatory indications, we included fistulae or perforation for following reasons: anastomotic leaks, diverticulitis, and inflammatory bowel disease (IBD).

Exclusion criteria included patients younger than 18 years of age and abandoned HR. However, all the attempted HRs were completed with a minimally invasive or open approach with no aborted procedure. None of the operations were performed with a transanal or robotic approach.

This retrospective study was carried out following the STROBE guidelines. The study was entered into the local audit register and complies with all ethical requirements (ID 3451).

Univariable analysis was performed using the chi-square test for categorical data, whereas continuous data were analysed with Student's *t*-test or Mann–Whitney *U* test depending on distribution. Multivariable analysis, such as logistic regression, was used in adjusted analysis for the relationship between variables for binomial data. Statistical significance was defined at *p* < 0.05. All statistical analysis was undertaken using *R* Studio Version 3.1.1 (R Foundation, Boston, Massachusetts, USA).

## 3. Results

Over a period of 7 years, 124 patients underwent HR; 60 were females (49.58%) and 61 were males (50.41%) with a mean age of 59.57 years (age range 20-84).

The indications for HP were divided into 5 groups (Table 1). Combined operations to HR were found in 27 patients (22.31%) and included abdominal wall repairs, oophorectomy/salpingectomy, and excision of enterocutaneous fistulae. The combined operations and converted procedure are summarized in Table 1. The 6 conventional laparoscopic cases were not analysed as a separate group because the number was too small to have a reliable result.

This study ties to address two different topics: the role of SILS in HR and factors affecting surgical complexity during reversal. This is to give a broad view of the issues involved in HR. We will describe them in this order.

As such, starting from the operative approaches, only two HP were performed laparoscopically (1.65%); instead, 49.58% (*n* = 60) of the reversals were minimally invasive. Six patients (4.95%) were treated with a conventional laparoscopic approach, 34 cases (28.09%) had a SILS procedure, and 20 (16.53%) required other trocars in addition to it. Twenty patients (33.33%) were converted because of dense adhesions and 2 (1.65%) for short rectal stump, with an overall rate of conversion of 36.66%.

Analysing all the conversion rate and SILS procedures over the 7-year period, there was no trend to show that attempted glove port reversals increased during the study period (*p* = 0.30) or that the frequency of conversions decreased (*p* = 0.68), as all the consultants had completed their learning curve before the data collection. The overall median operative time was 165 minutes (IQR 125-215). Excluding the cases with concomitant operations, SILS takes slightly longer than open technique (177 minutes vs 158 minutes), but without statistical significance (*p* = 0.06). Taking into account single-port procedures, there was a positive correlation (*p* = 0.01) between the length of the operation and the number of extra ports, showing an increase in surgical complexity.

Comparing all the minimally invasive procedures (SILS/laparoscopic operations) with the converted/open operations, there is a significant difference in hospital stay with a *p* = 0.007 (5 days vs 7 days). Postoperative complications (Clavien-Dindo 2-3-4-5) and unplanned reoperations did not differ between single port and open surgery (*p* = 0.83 and *p* = 0.42).

The mean time elapsed between HP and HR was 16.39 months for diverticulitis, 12.92 months for cancer patients, 10.66 months for Crohn's cases, and 19.06 months for postoperative complications. A protective loop ileostomy was formed during HR in 10 patients (8.26%) due to a low anastomosis or positive air-leak test. Patients with a short rectal stump were more likely to require a protective ileostomy (*p* = 0.008).

The most important operative findings causing operative complexity are the grade of adhesions and the length of the rectal stump. High grade of adhesions (grade 2 and 3) were found in 71.90% of the patients (*n* = 87) and a short rectal stump in 22.31% (*n* = 27). Worse adhesions showed to increase the risk of conversion (*p* < 0.001), prolong the length of the procedure (*p* < 0.001, with a mean time of 124 minutes for grade 0-1 and 204 minutes for grade 2-3), and increase the hospital stay (*p* = 0.05).

**Figure 1:**
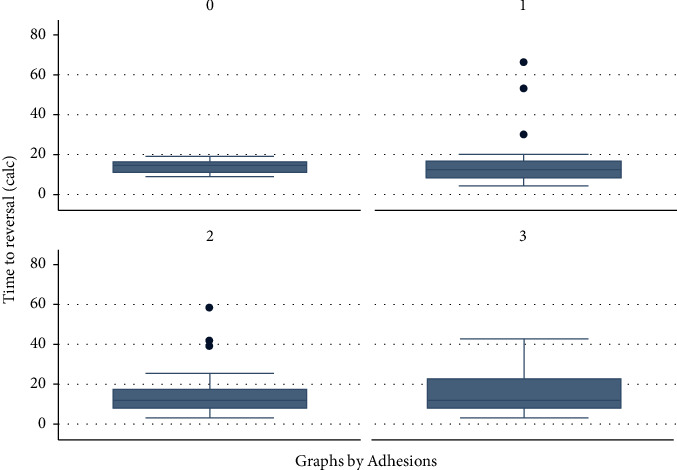
Time elapsed between HP and HR in the four grades of adhesions.

As shown in Figure 1, there is no difference in the time elapsed to reversal in the 4 tiers of adhesion severity, showing no association between early reversal and worse operative findings. However, HP for diverticulitis showed more adhesions when compared with the cancer group (*p* = 0.01).

Thirty patients (24.79%) had postoperative complications with a Clavien-Dindo 2 or worse, whereas major complications (Clavien-Dindo 3-4-5) occurred in 11 cases (9.09%). Only 1 death was recorded (mortality rate 0.82%), caused by a leak from an accidental enterotomy. Two patients had an anastomotic leak (1.65%).

Postoperative complication rate was not affected by grade of adhesions (*p* = 0.35), rectal stump length (*p* = 0.44), urgency of the original HP (*p* = 0.79), gender (*p* = 0.68), and time elapsed between resection and reversal (*p* = 0.17). However, age (*p* = 0.03), ASA score (*p* = 0.05), and operative length (*p* = 0.01) were independent factors increasing the risk of postoperative complications (Table 2).

## 4. Discussion

This study describes the outcomes of HR performed with single incision glove port, laparoscopic, and open technique. Furthermore, factors affecting postoperative morbidity and surgical complexity were defined using an ad hoc systematic classification of the grade of adhesions and rectal stump length. Throughout this simultaneous analysis of surgical techniques and factors influencing results, we tried to give an exhaustive idea of what an HR involves, allowing the surgeon to better select patients and plan the procedure.

A minimally invasive approach reduces postoperative morbidity in this frail group of patients due to less postoperative pain, early mobilisation, restoration of bowel function, and the return to a normal diet. In 1993, Anderson et al. published the first case of laparoscopic HR, and in 2011, Smith et al. described the first HR via stoma site, which takes advantage of the stoma opening for minimising the access trauma even further than conventional laparoscopic surgery [11–14]. The classic laparoscopic approach for HR has been widely described in literature and is considered to be safe with several advantages over the open surgery. Comparative studies showed a similar average operative time (171 for laparoscopic and 167 for open) and shorter mean length of stay (6 vs 11 days). Additionally, postoperative complications (16.4%) and mortality (0.7%) were lower. The laparoscopic conversion rate was between 5 and 25%, mostly due to adhesions (67.4%) and secondly to short stumps (7.2%) [2–7].

On the other hand, SILS through the stoma site has not had the same systematic analysis, and currently there are few related articles with limited cases. A recent systematic review (2020) analysed open, laparoscopic, and SILS procedures [15]. However, the number of articles taken into consideration for the SILS approach was only 4, as to show the lack of data on this topic.

The reported mean operative time ranges between 74 and 165 min, with a hospital stay of 3-8 days [16–18]. Choi et al. described 23 patients treated with SILS, with 1 aborted case and 4 postoperative complications including one anastomotic leak (4.5%) treated with resection and reanastomosis without faecal diversion [18]. Clermonts et al. and Thambi et al. compared SILS procedures to open ones and showed that the former had a shorter length of stay and less postoperative complications, including wound infections [8–19]. D'alessandro et al. carried out a case-controlled study between laparoscopic and SILS approach and concluded that the latter had a shorter operative time and hospital stay [20].

In our experience, the mean operative time (165 min), length of stay (5 days), major complication rate (9%), and mortality (0.8%) compared favourably with those reported in the literature for single port and laparoscopic surgery. The SILS operations had almost the same length as the open approach, whereas the hospital stay was slightly shorter. The overall risk of complications was low, and there was only one fatality. Despite this, it is difficult to compare the completed minimally invasive operations with the converted and open cases because there is a case selection bias towards more complex cases performed via an open approach. Even though a higher morbidity was expected in open cases, there was no difference in complications between the two groups. However, the anastomotic leak rate (1.65%) was remarkably lower when compared with the laparoscopic (3.6–12%) and single port cases (4.5%) described in the literature [1, 7, 14–21].

About one third of the minimally invasive cases were converted, with a higher overall rate (36.6%) compared to the laparoscopic data (5–25%) and the few SILS cases (4.3%) reported in the literature. This result can be attributed to the more complex technical approach related to SILS. Nonergonomic trocar placement makes adhesiolysis through the stoma site challenging. Several reported studies had an initial laparoscopic HP, whereas in our series most cases had open HP [7–18].

The mean time to reversal reported in the literature was 9 months which ensures the abdomen is more favourable with fewer severe adhesions, allows completion of adjuvant radio-chemotherapy and optimisation of clinical conditions and nutritional status. Almost all articles reported a shorter time to reversal when the initial indication for HP was due to diverticulitis in comparison with cancer, probably because any complication, especially infective ones like anastomotic leaks, may delay an adjuvant chemotherapy [1–23]. In our experience, the overall mean time to reversal was longer (15 months). Unexpectedly, cancer patients had a shorter time to reversal than those with diverticulitis which may be attributed to the more accurate and frequent follow-up schedule by the colorectal cancer nurses. In fact, none of the cancer patients that needed an HP was deemed suitable for a postoperative chemotherapy treatment, which could be the cause of a possible delay.

The timing of the reversal is controversial. Some authors argue that reversals performed less than 6 months after the initial operation have fewer postoperative complications, specifically anastomotic complications (5 times more frequent) [23–25]. Other studies recommend a minimum wait of 6 months because early operations are significantly associated with a longer length of stay and higher operative difficulty due to adhesions [4, 26, 27].

Our results showed no association between time to reversal and grade of adhesions, but it was still a major factor of the operative complexity and increased conversion rate, operative time, and length of stay. Patients with diverticulitis had more severe adhesions than cancer cases.

After 8–10 weeks from the HP, rectal atrophy is common, and short rectal stumps are difficult to visualise [7–14]. In our series, a short rectal stump does not influence the overall risk of conversion but does increase the rate of a defunctioning ileostomy.

Several studies, including a recent article by Whitney et al., have examined risk factors (albumin level <3.5 g/dL, obesity or BMI < 30, liver disease, and ASA score) predicting morbidity of HR [1–31]. In our series, age, longer operative time, and ASA score (none of our patients had an ASA score of 4) increased the rate of postoperative complications.

### 4.1. Limitations

We have a relatively large but retrospective and nonrandomised series. In addition to that, the number of open cases were almost the double of the SILS port when excluding the converted procedures.

We do not have any data about postoperative pain and the start of bowel function, which instead, could be favourably impacted by the SILS approach. This information was not always documented in the notes. Additionally, we have not analysed the postoperative quality of life in those groups.

Not all the patients were scoped before Hartmann's reversal to assess the length of the rectal stump. For this reason, we do not have a numerical value but we had to define the rectal stump as long or short in relation to the peritoneal reflection.

Unfortunately, access to the data of the overall operations performed in emergency was difficult and we were not able to know how many cases of HP were performed in those 7-year period. This would have allowed us to gather more information about the overall percentage of reverted patients and the clinical reasons for declining a colostomy closure. In addition, the database comprises patients until 2016, and the collection of data was stopped for technical reasons. A more extensive data collection would have allowed to find a higher number of HR procedures. Despite this, a good number of cases were included to allow a statistical analysis that would not be much altered by including more recent cases, as the learning curve of the operating consultants was already complete in 2010.

Our series does not include robotic or transanal HR, as our unit was not performing such procedures. These techniques may facilitate the approach to an hostile abdomen (in case of a transanal dissection) and reduce the technical challenges of the SILS operations (the articulation of robotic arms might be useful to overcome the instrument clashing in a limited space such as the glove port). Hopefully, in the future, other authors will be able to give us more information through large randomised trials.

## 5. Conclusion

Single port access through the stoma site is a safe and feasible approach for Hartmann's reversal procedures that offers additional cosmetic advantages without increasing morbidity and mortality rates in comparison to open and laparoscopic surgery. ASA score, long operative time, and age were recognised as the only independent factors affecting the postoperative morbidity.

## Figures and Tables

**Table 1:** Demographic data for glove port and open approach. Combined and converted operations were excluded.

	Glove port (*n* = 35)	Open (*n* = 64)	p
Age (median)	61.1 (48.2–73.3)	60.2 (50.8–67.3)	0.485
Sex (male)	20 (57)	32 (50)	0.463
ASA			0.983
1	4	13	—
2	26	36	—
3	5	15	—
Indication			
Cancer	9	14	—
Diverticulitis	19	32	—
Crohn's disease	0	3	—
Iatrogenic	5	11	—
Trauma	1	2	—
Volvulus	0	2	—
Thrombosis	1	0	—
Inflammatory	25 (71.4%)	44 (68.8%)	0.782
Emergency	31 (88.6%)	49 (76.6%)	0.147
Time to reversal (months)	11.5 (8–17)	11 (8–18)	0.582
Adhesions 0/1	18 (51.4%)	53 (82.8%)	0.001
Length of rectal stump			0.087
Long	30	45	—
Short	5	19	—
Operation time (min)	165 (125–210)	165 (130–210)	0.901
Protective loop ileostomy	1	8	0.153
Complications (≥ CD2)	7 (20%)	18 (28.1%)	0.471
Complications (≥ CD3)	5 (14.3%)	4 (6.3%)	0.272
Reoperation	3 (8.6%)	2 (3.1%)	0.342
Mortality	0	1 (1.6%)	0.871

**Table 2:** Risk factors for complications (Clavien-Dindo 2 or higher).

	Odds ratio	95% CI	p
Age (y)	1.045	1.007–1.083	0.018
Gender	1.22	0.53–2.79	0.636
ASA			
1	7.89	1.09–59.3	0.032
2	8.00	1.01–63.3	0.049
3	8.75	0.97–78.7	0.053
Inflammatory indication	0.54	0.23–1.28	0.160
Emergency	1.32	0.45–3.91	0.617
Time to reversal (m)	1.03	1.00–1.07	0.069
Adhesions	2.34	0.81–6.73	0.115
Length of stump	1.38	0.53–3.58	0.510
Operative time (min)	8.74	0.97–78.7	0.052

## Data Availability

Data are available on request.
